# 3-Benzyl­idene-6-methoxy­chroman-4-one

**DOI:** 10.1107/S1600536808031541

**Published:** 2008-10-09

**Authors:** T. Augustine, Scholastica Mary Vithiya, V. Ramkumar, Charles C. Kanakam

**Affiliations:** a Department of Chemistry, Presidency College, Chennai, Tamil Nadu, India; b Department of Chemistry, Auxilium College, Vellore, Tamil Nadu, India; c Department of Chemistry, Indian Institute of Technology Madras, Chennai 36, Tamil Nadu, India; d Department of Chemistry, Valliammai Engineering College, SRM Nagar, Chennai, Tamil Nadu, India

## Abstract

In the title compound, C_17_H_14_O_3_, the dihedral angle between the phenyl ring and the benzene ring of the chromanone moiety is 67.78 (3)°. The six-membered heterocyclic ring of the chromanone moiety adopts a half-chair conformation. The structure is stabilized by weak inter­molecular C—H⋯O inter­actions that link the mol­ecules into inversion dimers.

## Related literature

For background literature, see: Finch & Tamm (1970 ▶); Geen *et al.*(1996 ▶); Tietze & Gerlitzer (1997 ▶); Cremer & Pople (1975 ▶). For a related structure, see: Suresh *et al.* (2007 ▶).
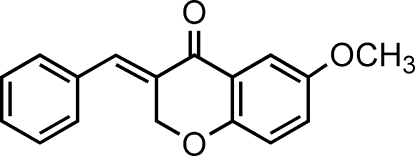


## Experimental

### 

#### Crystal data

C_17_H_14_O_3_
*M* *_r_* = 266.28
Triclinic, 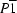

*a* = 7.2678 (2) Å
*b* = 8.3151 (2) Å
*c* = 11.7999 (4) Å
α = 95.964 (1)°
β = 103.828 (1)°
γ = 104.042 (1)°
*V* = 661.74 (3) Å^3^
*Z* = 2
Mo *K*α radiation
μ = 0.09 mm^−1^
*T* = 298 (2) K
0.45 × 0.42 × 0.38 mm

#### Data collection

Bruker APEXII CCD area-detector diffractometer
Absorption correction: multi-scan (*SADABS*; Bruker, 1999 ▶) *T*_min_ = 0.960, *T*_max_ = 0.966
8868 measured reflections
3041 independent reflections
2404 reflections with *I* > 2σ(*I*)
*R*_int_ = 0.018

#### Refinement

*R*[*F* ^2^ > 2σ(*F* ^2^)] = 0.039
*wR*(*F* ^2^) = 0.116
*S* = 1.04
3041 reflections
186 parameters
H atoms treated by a mixture of independent and constrained refinement
Δρ_max_ = 0.24 e Å^−3^
Δρ_min_ = −0.23 e Å^−3^

### 

Data collection: *APEX2* (Bruker, 2004 ▶); cell refinement: *APEX2* (Bruker, 2004 ▶); data reduction: *SAINT-Plus* (Bruker, 2004 ▶); program(s) used to solve structure: *SHELXS97* (Sheldrick, 2008 ▶); program(s) used to refine structure: *SHELXL97* (Sheldrick, 2008 ▶); molecular graphics: *ORTEP-3* (Farrugia, 1997 ▶); software used to prepare material for publication: *SHELXL97* and *PLATON* (Spek, 2003 ▶).

## Supplementary Material

Crystal structure: contains datablocks global, I. DOI: 10.1107/S1600536808031541/fl2222sup1.cif

Structure factors: contains datablocks I. DOI: 10.1107/S1600536808031541/fl2222Isup2.hkl

Additional supplementary materials:  crystallographic information; 3D view; checkCIF report

## Figures and Tables

**Table 1:** Hydrogen-bond geometry (Å, °)

*D*—H⋯*A*	*D*—H	H⋯*A*	*D*⋯*A*	*D*—H⋯*A*
C6—H6⋯O1^i^	0.93	2.53	3.4293 (18)	163

Symmetry code: (i) 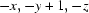.

**Fig. 1.**
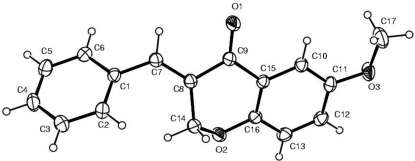
ORTEP of the molecule with atoms represented as 30% probability ellipsoids.

## supplementary crystallographic information

### Comment

The chroman-4-one (2,3-dihydro-4-oxo-4*H*-1-benzopyran) ring system occupies an important position among oxygen heterocyclics and features in a wide variety of compounds of biological and medicinal interest (Finch & Tamm, 1970). Many biologically active natural products containing a chroman ring system have been synthesized *via* 2-substituted chroman-4-one intermediates including alpha-tocopherol (vitamin E) (Geen *et al.*, 1996). 3-arylidene-4-chromanones have also been isolated as natural products belonging to the class of compounds called homoisoflavonoids(Tietze & Gerlitzer, 1997).

The geometric parameters in the title compound agree with values reported for a similar structure (Suresh *et al.*, 2007). The dihedral angle between the benzene ring of the chromanone moiety and the phenyl ring is 67.78 (3)°. The Chromanone moiety is fused with a six membered heterocyclic ring and the study of torsion angles, asymmetry parameters and least-square plane calculations shows that the chromanone adopts a half chair conformation with a deviation of C14 from the C8/C9/C15/C16/O2 plane by 0.616 (4) Å, Q_2_= 0.4053 (14) Å, Q_3_= -0.2052 (13)Å, and  Q_T_=0.4543 (14)Å (Cremer &Pople, (1975). The structure is stabilized by weak intermolecular C—H···O interaction that link the molecules into pairs around a center (Table 1). No other short intermolecular interactions were found.

### Experimental

Methyl-(2*Z*)-2-bromo methyl-3-aryl prop-2-enoate (0.006 mole, 1.53 g) was treated with 4-methoxy phenol (0.006 mole, 0.9 ml) in the presence of potassium carbonate in acetone at reflux temperature for 3 hrs. The pure ester, 3-aryl-2-(4-methoxy)-phenoxymethylprop-2-enoate was obtained after purifying it using silica gel and column chromatography (3% ethyl acetate - hexane). Hydrolysis of this ester was carried out with KOH in aqueous 1,4–dioxane at room temperature. The reaction mixture was acidified and the precipitated acid was purified by recrystalization. Finally the acid was treated with triflouroacetic anhydride and the reaction mixture was refluxed in dichloro- methane for 1 hr. It was further purified by column chromatography (silica gel-3% ethyl acetate - hexane) and the crystals used for data collection were obtained by slow evaporation from methanol.

### Refinement

H atoms were positioned geometrically and refined using riding model,with C—H = 0.93 Å and *U*_iso_(H) = 1.2*U*_eq_(C) for aromatic C—H, C—H = 0.97 Å and *U*_iso_(H) = 1.2*U*_eq_(C) for CH2, C—H = 0.96 Å and *U*_iso_(H) = 1.5*U*_iso_(C) for CH3.

### Crystal data

**Table:**

C_17_H_14_O_3_	*Z* = 2
*M**_r_* = 266.28	*F*(000) = 280
Triclinic, *P*1	*D*_x_ = 1.336 Mg m^−^^3^
Hall symbol: -p 1	Mo *K*α radiation, λ = 0.71073 Å
*a* = 7.2678 (2) Å	Cell parameters from 4851 reflections
*b* = 8.3151 (2) Å	θ = 2.6–28.3°
*c* = 11.7999 (4) Å	µ = 0.09 mm^−^^1^
α = 95.964 (1)°	*T* = 298 K
β = 103.828 (1)°	Block, colourless
γ = 104.042 (1)°	0.45 × 0.42 × 0.38 mm
*V* = 661.74 (3) Å^3^	

### Data collection

**Table:**

Bruker APEXII CCD area-detector diffractometer	3041 independent reflections
Radiation source: fine-focus sealed tube	2404 reflections with *I* > 2σ(*I*)
graphite	*R*_int_ = 0.018
φ and ω scans	θ_max_ = 28.3°, θ_min_ = 2.6°
Absorption correction: multi-scan (SADABS; Bruker, 1999)	*h* = −8→9
*T*_min_ = 0.960, *T*_max_ = 0.966	*k* = −10→11
8868 measured reflections	*l* = −15→15

### Refinement

**Table:**

Refinement on *F*^2^	Primary atom site location: structure-invariant direct methods
Least-squares matrix: full	Secondary atom site location: difference Fourier map
*R*[*F*^2^ > 2σ(*F*^2^)] = 0.039	Hydrogen site location: inferred from neighbouring sites
*wR*(*F*^2^) = 0.116	H atoms treated by a mixture of independent and constrained refinement
*S* = 1.04	*w* = 1/[σ^2^(*F*_o_^2^) + (0.0554*P*)^2^ + 0.1403*P*] where *P* = (*F*_o_^2^ + 2*F*_c_^2^)/3
3041 reflections	(Δ/σ)_max_ < 0.001
186 parameters	Δρ_max_ = 0.25 e Å^−^^3^
0 restraints	Δρ_min_ = −0.23 e Å^−^^3^

### Special details

**Table:**

Geometry. All e.s.d.'s (except the e.s.d. in the dihedral angle between two l.s. planes)are estimated using the full covariance matrix. The cell e.s.d.'s are takeninto account individually in the estimation of e.s.d.'s in distances, anglesand torsion angles; correlations between e.s.d.'s in cell parameters are onlyused when they are defined by crystal symmetry. An approximate (isotropic)treatment of cell e.s.d.'s is used for estimating e.s.d.'s involving l.s. planes.
Refinement. Refinement of *F*^2^ against ALL reflections. The weighted *R*-factor *wR* andgoodness of fit *S* are based on *F*^2^, conventional *R*-factors *R* are basedon *F*, with *F* set to zero for negative *F*^2^. The threshold expression of*F*^2^ > σ(*F*^2^) is used only for calculating *R*-factors(gt) *etc*. and isnot relevant to the choice of reflections for refinement. *R*-factors basedon *F*^2^ are statistically about twice as large as those based on *F*, and *R*-factors based on ALL data will be even larger.

### Fractional atomic coordinates and isotropic or equivalent isotropic displacement parameters (Å^2^)

**Table:**

	*x*	*y*	*z*	*U*_iso_*/*U*_eq_	
C1	−0.38800 (18)	0.42099 (15)	0.12980 (11)	0.0357 (3)	
C2	−0.49675 (19)	0.45875 (16)	0.20631 (12)	0.0413 (3)	
H2	−0.4480	0.5584	0.2610	0.050*	
C3	−0.6764 (2)	0.34969 (18)	0.20189 (13)	0.0469 (3)	
H3	−0.7484	0.3771	0.2527	0.056*	
C4	−0.7491 (2)	0.20056 (18)	0.12250 (14)	0.0499 (3)	
H4	−0.8689	0.1265	0.1205	0.060*	
C5	−0.6433 (2)	0.16181 (18)	0.04615 (13)	0.0526 (4)	
H5	−0.6916	0.0606	−0.0069	0.063*	
C6	−0.4668 (2)	0.27146 (17)	0.04756 (12)	0.0448 (3)	
H6	−0.3995	0.2458	−0.0066	0.054*	
C7	−0.19833 (18)	0.53387 (16)	0.12988 (12)	0.0384 (3)	
C8	−0.04981 (18)	0.62292 (15)	0.22311 (11)	0.0366 (3)	
C9	0.12813 (18)	0.73207 (16)	0.20106 (11)	0.0375 (3)	
C10	0.47891 (18)	0.89529 (15)	0.29540 (11)	0.0370 (3)	
H10	0.4781	0.9476	0.2294	0.044*	
C11	0.64996 (18)	0.92856 (15)	0.38626 (11)	0.0390 (3)	
C12	0.65136 (19)	0.84577 (17)	0.48305 (12)	0.0439 (3)	
H12	0.7679	0.8659	0.5429	0.053*	
C13	0.4833 (2)	0.73495 (18)	0.49135 (12)	0.0439 (3)	
H13	0.4862	0.6800	0.5562	0.053*	
C14	−0.03927 (19)	0.62083 (18)	0.35174 (11)	0.0427 (3)	
H14A	−0.0509	0.7271	0.3874	0.051*	
H14B	−0.1486	0.5319	0.3584	0.051*	
C15	0.30664 (17)	0.78222 (14)	0.30322 (10)	0.0336 (3)	
C16	0.30814 (18)	0.70497 (15)	0.40210 (11)	0.0362 (3)	
C17	0.8268 (3)	1.1358 (3)	0.29888 (18)	0.0764 (6)	
H17A	0.8124	1.0644	0.2261	0.115*	
H17B	0.9493	1.2225	0.3186	0.115*	
H17C	0.7195	1.1861	0.2896	0.115*	
O1	0.13097 (14)	0.77364 (14)	0.10531 (9)	0.0566 (3)	
O2	0.14409 (13)	0.59397 (12)	0.41471 (8)	0.0454 (2)	
O3	0.82540 (14)	1.03993 (13)	0.38987 (9)	0.0540 (3)	
H7	−0.175 (2)	0.5435 (19)	0.0546 (15)	0.051 (4)*	

### Atomic displacement parameters (Å^2^)

**Table:**

	*U*^11^	*U*^22^	*U*^33^	*U*^12^	*U*^13^	*U*^23^
C1	0.0311 (6)	0.0373 (6)	0.0357 (6)	0.0073 (5)	0.0046 (5)	0.0092 (5)
C2	0.0344 (6)	0.0382 (6)	0.0475 (7)	0.0079 (5)	0.0088 (5)	0.0016 (5)
C3	0.0365 (7)	0.0516 (8)	0.0538 (8)	0.0108 (6)	0.0158 (6)	0.0100 (6)
C4	0.0349 (7)	0.0470 (7)	0.0587 (9)	−0.0018 (6)	0.0082 (6)	0.0122 (6)
C5	0.0497 (8)	0.0431 (7)	0.0504 (8)	−0.0009 (6)	0.0048 (6)	−0.0030 (6)
C6	0.0426 (7)	0.0480 (7)	0.0384 (7)	0.0072 (6)	0.0091 (5)	0.0016 (5)
C7	0.0339 (6)	0.0423 (6)	0.0378 (6)	0.0081 (5)	0.0092 (5)	0.0087 (5)
C8	0.0310 (6)	0.0403 (6)	0.0380 (6)	0.0084 (5)	0.0097 (5)	0.0074 (5)
C9	0.0321 (6)	0.0423 (6)	0.0353 (6)	0.0069 (5)	0.0071 (5)	0.0079 (5)
C10	0.0331 (6)	0.0381 (6)	0.0375 (6)	0.0086 (5)	0.0064 (5)	0.0066 (5)
C11	0.0297 (6)	0.0386 (6)	0.0444 (7)	0.0081 (5)	0.0059 (5)	0.0025 (5)
C12	0.0347 (7)	0.0539 (8)	0.0385 (7)	0.0150 (6)	0.0006 (5)	0.0039 (6)
C13	0.0413 (7)	0.0551 (8)	0.0352 (6)	0.0154 (6)	0.0067 (5)	0.0111 (5)
C14	0.0321 (6)	0.0548 (8)	0.0392 (7)	0.0076 (6)	0.0114 (5)	0.0059 (6)
C15	0.0298 (6)	0.0357 (6)	0.0334 (6)	0.0087 (5)	0.0067 (5)	0.0032 (4)
C16	0.0334 (6)	0.0401 (6)	0.0349 (6)	0.0101 (5)	0.0100 (5)	0.0047 (5)
C17	0.0437 (9)	0.0871 (13)	0.0821 (13)	−0.0091 (9)	0.0049 (8)	0.0372 (10)
O1	0.0392 (5)	0.0764 (7)	0.0416 (5)	−0.0049 (5)	0.0035 (4)	0.0223 (5)
O2	0.0369 (5)	0.0580 (6)	0.0403 (5)	0.0077 (4)	0.0104 (4)	0.0173 (4)
O3	0.0305 (5)	0.0574 (6)	0.0614 (6)	−0.0006 (4)	−0.0003 (4)	0.0152 (5)

### Geometric parameters (Å, °)

**Table:**

C1—C2	1.3932 (18)	C10—C11	1.3805 (17)
C1—C6	1.3978 (17)	C10—C15	1.4007 (17)
C1—C7	1.4671 (17)	C10—H10	0.9300
C2—C3	1.3830 (19)	C11—O3	1.3704 (15)
C2—H2	0.9300	C11—C12	1.3927 (19)
C3—C4	1.378 (2)	C12—C13	1.371 (2)
C3—H3	0.9300	C12—H12	0.9300
C4—C5	1.377 (2)	C13—C16	1.3938 (17)
C4—H4	0.9300	C13—H13	0.9300
C5—C6	1.377 (2)	C14—O2	1.4434 (16)
C5—H5	0.9300	C14—H14A	0.9700
C6—H6	0.9300	C14—H14B	0.9700
C7—C8	1.3412 (17)	C15—C16	1.3883 (17)
C7—H7	0.950 (16)	C16—O2	1.3693 (15)
C8—C9	1.4846 (18)	C17—O3	1.403 (2)
C8—C14	1.5036 (18)	C17—H17A	0.9600
C9—O1	1.2187 (15)	C17—H17B	0.9600
C9—C15	1.4818 (16)	C17—H17C	0.9600
			
C2—C1—C6	118.20 (12)	O3—C11—C10	124.75 (12)
C2—C1—C7	122.70 (11)	O3—C11—C12	115.45 (11)
C6—C1—C7	119.07 (12)	C10—C11—C12	119.80 (12)
C3—C2—C1	120.72 (12)	C13—C12—C11	120.93 (12)
C3—C2—H2	119.6	C13—C12—H12	119.5
C1—C2—H2	119.6	C11—C12—H12	119.5
C4—C3—C2	120.26 (13)	C12—C13—C16	119.70 (12)
C4—C3—H3	119.9	C12—C13—H13	120.1
C2—C3—H3	119.9	C16—C13—H13	120.1
C5—C4—C3	119.63 (13)	O2—C14—C8	111.15 (10)
C5—C4—H4	120.2	O2—C14—H14A	109.4
C3—C4—H4	120.2	C8—C14—H14A	109.4
C6—C5—C4	120.64 (13)	O2—C14—H14B	109.4
C6—C5—H5	119.7	C8—C14—H14B	109.4
C4—C5—H5	119.7	H14A—C14—H14B	108.0
C5—C6—C1	120.49 (13)	C16—C15—C10	120.07 (11)
C5—C6—H6	119.8	C16—C15—C9	119.69 (11)
C1—C6—H6	119.8	C10—C15—C9	119.96 (11)
C8—C7—C1	128.33 (12)	O2—C16—C15	122.60 (11)
C8—C7—H7	115.3 (9)	O2—C16—C13	117.53 (11)
C1—C7—H7	116.4 (9)	C15—C16—C13	119.83 (12)
C7—C8—C9	118.66 (11)	O3—C17—H17A	109.5
C7—C8—C14	126.65 (12)	O3—C17—H17B	109.5
C9—C8—C14	114.65 (10)	H17A—C17—H17B	109.5
O1—C9—C15	121.81 (11)	O3—C17—H17C	109.5
O1—C9—C8	123.09 (11)	H17A—C17—H17C	109.5
C15—C9—C8	115.06 (11)	H17B—C17—H17C	109.5
C11—C10—C15	119.58 (12)	C16—O2—C14	113.85 (10)
C11—C10—H10	120.2	C11—O3—C17	117.35 (11)
C15—C10—H10	120.2		
			
C6—C1—C2—C3	0.93 (19)	C11—C12—C13—C16	−0.3 (2)
C7—C1—C2—C3	179.01 (12)	C7—C8—C14—O2	129.37 (13)
C1—C2—C3—C4	0.9 (2)	C9—C8—C14—O2	−48.50 (15)
C2—C3—C4—C5	−1.0 (2)	C11—C10—C15—C16	0.33 (18)
C3—C4—C5—C6	−0.7 (2)	C11—C10—C15—C9	−173.63 (11)
C4—C5—C6—C1	2.6 (2)	O1—C9—C15—C16	−166.84 (12)
C2—C1—C6—C5	−2.7 (2)	C8—C9—C15—C16	10.83 (17)
C7—C1—C6—C5	179.18 (12)	O1—C9—C15—C10	7.14 (19)
C2—C1—C7—C8	41.2 (2)	C8—C9—C15—C10	−175.19 (10)
C6—C1—C7—C8	−140.74 (14)	C10—C15—C16—O2	179.75 (11)
C1—C7—C8—C9	−178.86 (12)	C9—C15—C16—O2	−6.28 (18)
C1—C7—C8—C14	3.3 (2)	C10—C15—C16—C13	−2.59 (18)
C7—C8—C9—O1	16.3 (2)	C9—C15—C16—C13	171.39 (11)
C14—C8—C9—O1	−165.66 (13)	C12—C13—C16—O2	−179.65 (11)
C7—C8—C9—C15	−161.35 (11)	C12—C13—C16—C15	2.57 (19)
C14—C8—C9—C15	16.71 (16)	C15—C16—O2—C14	−27.22 (16)
C15—C10—C11—O3	−178.13 (11)	C13—C16—O2—C14	155.06 (11)
C15—C10—C11—C12	1.94 (18)	C8—C14—O2—C16	53.73 (14)
O3—C11—C12—C13	178.09 (12)	C10—C11—O3—C17	5.0 (2)
C10—C11—C12—C13	−2.0 (2)	C12—C11—O3—C17	−175.11 (15)

### Hydrogen-bond geometry (Å, °)

**Table:**

*D*—H···*A*	*D*—H	H···*A*	*D*···*A*	*D*—H···*A*
C6—H6···O1^i^	0.93	2.53	3.4293 (18)	163

Symmetry codes: (i) −*x*, −*y*+1, −*z*.
